# Domain-less defenders: evolutionary innovations and agricultural promise of noncanonical NLRs

**DOI:** 10.1016/j.abiote.2026.100048

**Published:** 2026-04-03

**Authors:** Zichen Liu, Man Hu, Guozhi Bi

**Affiliations:** a State Key Laboratory of Plant Environmental Resilience, Frontiers Science Center for Molecular Design Breeding, College of Biological Sciences, China Agricultural University, Beijing, 100193, China; b State Key Laboratory of Plant Genomics, Institute of Genetics and Developmental Biology, Chinese Academy of Sciences, Beijing, 100101, China

**Keywords:** Noncanonical NLRs, NL proteins, Sorghum aphid resistance, Signaling activation

## Abstract

Noncanonical nucleotide-binding leucine-rich repeat (NLR) genes, which lack typical N-terminal domains, are abundant in plants but their functions remain poorly understood. Two recent studies have cloned members of this family and offer clues into the role of this family in plant immunity. Independently, these workers cloned two noncanonical NLR genes, *RMES1A* and *RMES1B*, that confer resistance to the aphid *Melanaphis sorghi* in sorghum (*Sorghum bicolor*). Furthermore, their cognate insect effector MsEF1, a phosphatase-like protein, was identified. These studies establish that NLRs lacking clear functional domains can mediate insect resistance and provide a new genetic resource for engineering pest-resilient crops.

## 1 Introduction

Phloem-feeding insects pose a severe threat to global crop production on two levels. First, they drain essential nutrients from plant vascular systems; second, they also serve as efficient vectors for plant pathogens. To defend against these pests, plants employ nucleotide-binding leucine-rich repeat (NLR) immune receptors. In rice (*Oryza sativa*), the brown planthopper (BPH) is a major constraint on yields; multiple resistance genes to BPH have been identified, including canonical *NLR* genes (*BPH1*, *2*, *7*, *9*, *10*, *14*, *18*, *21*, and *26*) and noncanonical *NLR* genes (*BPH6*, *30*, and *40*) [[1], [2], [3]]. Beyond rice, well-characterized examples of NLR-mediated insect resistance include the *Meloidogyne incognita resistance 1.2* (*Mi-1.2*) gene in tomato (*Solanum lycopersicum*) conferring resistance to potato aphids [4], the *Virus aphid transmitted* (*Vat*) locus of melon (*Cucumis melo*) protecting against cotton aphids [5], and the *Resistance to Aphis glycines 1* (*Rag1*) gene of soybean (*Glycine max*) conferring resistance against soybean aphids [6].

Since 2013, the sorghum aphid *Melanaphis sorghi* (*MES*) has emerged as a devastating pest in sorghum (*Sorghum bicolor*) fields, spreading pandemically across major sorghum-growing regions. While genetic mapping over the past decade has identified the *Resistance to M. sorghi 1* (*RMES1*) locus as a major quantitative trait locus (QTL) governing sorghum aphid resistance, the underlying gene(s) and mechanisms of this resistance have remained elusive. Recently, two independent groups resolved the *RMES1* locus into the two genes *RMES1A* and *RMES1B* using forward genetics and discovered they are noncanonical *NLR* genes mediating sorghum resistance to *MES* [7,8]. Functional and evolutionary characterization of these genes advances our understanding of the biological functions of noncanonical NLR proteins in plant immunity.

## 2 The RMES1 system: a new paradigm for NL protein functions

**Fig. 1:**
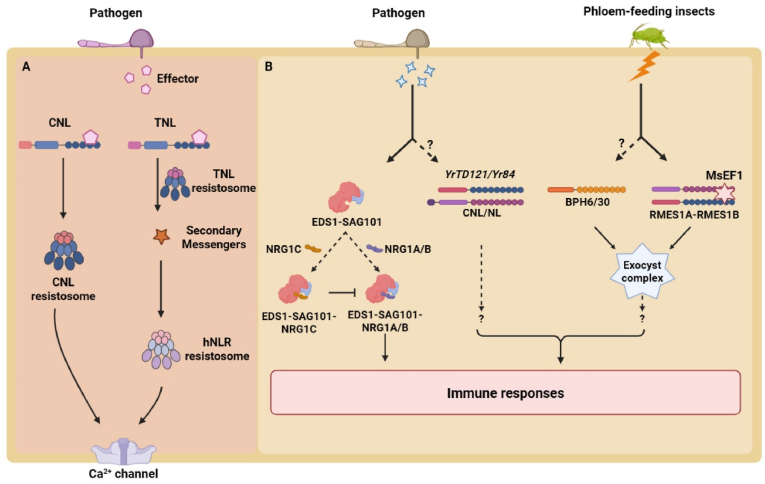
Plant immune mechanisms orchestrated by canonical NLRs and NLs. **A** Canonical NLR-mediated immune responses to pathogens. Upon recognition of pathogen effectors, CNLs oligomerize into resistosomes that directly function as Ca^2+^ channels to initiate immune signaling cascades; TNLs also assemble into resistosomes. Their TIR domains produce secondary messengers, which subsequently activate helper NLR resistosomes that in turn drive Ca^2+^ channel–dependent immune transduction. **B** Regulation of immunity by and defensive functions of NLs. The NL protein NRG1C associates with the EDS1–SAG101 complex to fine-tune immune homeostasis. Some NLs act cooperatively with canonical NLRs to mediate resistance against wheat stripe rust. Notably, the sorghum NL proteins RMES1A and RMES1B function synergistically to confer robust resistance against the aphid *Melanaphis sorghi*. Similar to rice NLs (e.g., BPH6 and BPH30), RMES1A and RMES1B localize to and associate with the exocyst complex, a subcellular distribution that diverges from that of most canonical NLRs. Figure created with BioRender (BioRender.com).

Canonical plant NLRs exhibit a tripartite architecture, comprising a variable N-terminal signaling domain, a central nucleotide-binding (NB) adaptor domain shared by APAF-1, R proteins, and CED-4 (NB-ARC), and a C-terminal leucine-rich repeat (LRR) domain. Based on variation in their N-terminal domains, canonical NLR proteins can be classified into three main types: CNLs (CC-NLRs), TNLs (Toll/interleukin-1 receptor [TIR]-NLRs), and RNLs (RESISTANCE TO POWDERY MILDEW 8 [RPW8]-like NLRs). The LRR domains of these receptors are primarily responsible for pathogen detection, with some NLRs also requiring integrated domains (IDs) for effector binding. Upon effector recognition, canonical NLRs oligomerize into multimeric complexes known as resistosomes that initiate downstream immune signaling. In particular, CNL resistosomes form calcium ion channels via their CC domains, while TNL resistosomes activate NAD^+^ glycohydrolase (NADase) activity through their TIR domains, producing small-molecule metabolites (e.g., pRib-AMP, pRib-ADP, ADPr-ATP, and di-ADPR). These small molecules selectively activate helper RNLs (e.g., ACTIVATED DISEASE RESISTANCE 1 [ADR1] and N REQUIREMENT GENE 1 [NRG1]), which in turn form calcium channels (Fig. 1A) [9]. Although many aspects of canonical NLR signaling have been extensively elucidated in recent years, this paradigm is insufficient to encompass the full diversity of the NLR superfamily.

NL (NB-ARC-LRR) proteins are an intriguing class of noncanonical NLRs; they have neither an N-terminal CC nor a TIR domain [10]. Although their architecture appears incomplete, NL genes are remarkably abundant in plant genomes and represent the second-largest group within the plant NLR gene repertoire behind canonical NLR genes. To date, the biological roles of most NL proteins remain elusive, as only a few have been functionally characterized. For example, the RNL protein NRG1C, a member of the NL subfamily, acts as a negative regulator that suppresses autoimmunity and maintains immune homeostasis [[11], [12], [13]]. Furthermore, the rice genes *BPH6*, *BPH30*, and *BPH40* encode NL proteins that confer resistance to BPH [2,3]. NLs can also function cooperatively with canonical NLRs, as demonstrated in wheat (*Triticum aestivum*), where they contribute to stripe rust resistance mediated by *YrTD121* and *Yr84* originating from wild emmer wheat (*Triticum turgidum* ssp. *dicoccoides*) [14,15]. Recently, VanGessel et al. and Lei et al. identified two NL genes, *RMES1A* and *RMES1B*, which together mediate sorghum resistance to *MES* (Fig. 1B) [7,8]. Furthermore, Lei et al. identified the *MES* effector MsEF1, which associates with the LRR domains of RMES1A and RMES1B [8]. This interaction occurs at the surface of vesicles decorated by the exocyst complex, where RMES1A and RMES1B reside. Functionally, RMES1A and RMES1B are both required and work in concert to confer resistance, a mode of action distinct from the autonomous action of NLs or NL–canonical NLR partnerships [7,8]. This cooperative requirement between two NLs may indicate a previously unknown configuration for immune activation, broadening functional diversity within the NLR repertoire.

Distinct regulatory modes have emerged among functionally characterized NL proteins. For instance, the NL protein NRG1C competes with its helper NLR partner NRG1A for binding to the ENHANCED DISEASE SUSCEPTIBILITY 1 (EDS1)–SENESCENCE-ASSOCIATED GENE 101 (SAG101) complex. NRG1C occupies this critical signaling node but does not trigger downstream activation, effectively suppressing immune responses [12,13]. By contrast, evidence from wheat suggests that certain NLs may associate with canonical NLRs, as seen in stripe rust resistance mediated by *YrTD121* and *Yr84*. In such partnerships, NLs are thought to interact with the signaling domains of their canonical partners to activate immune responses [14,15]. A key question prompted by the study of VanGessel et al. is how two NLs, such as RMES1A and RMES1B, might coordinately induce resistance in the absence of an apparent canonical signaling partner. VanGessel et al. showed that these two RMES1 proteins do activate canonical immune signaling pathways, including salicylic acid signaling, oxylipin biosynthesis via 9-lipoxygenases, and WRKY-regulated transcription [7]. These observations support the view that NLs can engage core downstream NLR signaling networks, even though they lack canonical activation domains themselves.

## 3 NL proteins: more than just evolutionary relics

Given their demonstrated capacity to activate immunity, the prevalence of NL genes in plant genomes raises a fundamental evolutionary question: are they primarily non-functional relics resulting from the loss of sequences encoding the N-terminal domains of NLRs during evolution, or have they been shaped and maintained by selective pressures?

The evolutionary significance of NL proteins is first reflected in the diversification of helper NLR families. The truncated suppressor NRG1C is functionally conserved among *Brassicaceae* species and with its homolog NRG2 in *Nicotiana benthamiana*, revealing a cross-species conserved strategy for immune regulation [11]. Notably, the pattern of domain loss illustrated by NL genes has occurred independently in monocotyledonous species, as their members of the ADR1 family have lost the RPW8 domain [16]. These findings indicate that NL-type RNLs are adaptive products of structural innovation, enabling precise fine-tuning of the plant immune system.

Phylogenetic and synteny analyses established that *RMES1* belongs to an ancient lineage within the Poaceae. Indeed, this locus sits within a syntenic block across major grasses such as purple false brome (*Brachypodium distachyon*), rice, and green foxtail (*Setaria viridis*). Notably, the rice ortholog at this conserved syntenic locus is *BPH40* [3]. This independent recruitment of orthologous NL genes for similar defense functions in different grass species is indicative of adaptive evolution. Furthermore, pangenome analysis revealed extensive copy-number variation at this locus. The resistant haplotype at this locus, containing both *RMES1A* and *RMES1B*, is rare in global sorghum landraces and is primarily present in accessions from East Africa and Yemen, suggesting it has been maintained as standing genetic variation rather than being a recent innovation. This pre-existing adaptation, likely honed by past historical pest pressure in regions like Sudan, provided the large-effect variant necessary for combatting the recent global aphid outbreak. Thus, ancient evolutionary ancestry and preserved genetic diversity in NL loci can serve as a crucial reservoir, facilitating rapid adaptive responses to emerging selective pressures.

## 4 Concluding remarks and future perspectives

While these two studies offered valuable cues into the molecular mechanisms of NLs, they also raised several questions for future investigation. A primary question is how noncanonical NLRs like RMES1A and RMES1B, which lack a canonical N-terminal signaling domain and possess a non-functional P-loop in their NB-ARC region, can successfully activate well-established defense pathways? Do they trigger defense signaling through canonical NLRs? Alternatively, might they interact with as yet unidentified co-receptors or adaptor proteins to bypass the traditional requirement for a CC or TIR domain? Furthermore, similar to the rice NL proteins BPH6 and BPH30, RMES1A and RMES1B associate with vesicles decorated by the exocyst complex, defining a subcellular compartment that is distinct from that of most canonical NLRs. Is this specific compartmentalization essential for their function? The current findings represent only the initial steps in unraveling the functional landscape of NL proteins. Most of the NL proteins encoded by plant genomes remain molecular orphans, and their biological functions and molecular mechanisms are completely unknown.

Beyond understanding their native function, these noncanonical NLRs present a compelling opportunity for bioengineering. Indeed, engineering canonical NLRs for expanded disease resistance is often hindered by their propensity to auto-activate, which can compromise plant growth [17]. Given that NL proteins like RMES1A and RMES1B naturally lack the signaling domains that are responsible for auto-activity, they may offer a more stable and tractable framework for engineering durable and specific resistance in crops, potentially circumventing a significant bottleneck in the development of synthetic immune receptors.

## CRediT authorship contribution statement

**Zichen Liu:** Writing – original draft. **Man Hu:** Writing – review & editing, Writing – original draft. **Guozhi Bi:** Writing – review & editing, Supervision, Funding acquisition, Conceptualization.

## Declaration of competing interest

No interests are declared.

## Data Availability

Data sharing is not applicable to this article as no datasets were generated or analyzed during the current study.
